# Successful perforation management during esophageal endoscopic submucosal dissection using the stent‐anchoring method: A case report

**DOI:** 10.1002/deo2.70114

**Published:** 2025-04-21

**Authors:** Mai Utsumi, Taro Iwatsubo, Kazuki Takayama, Shun Sasaki, Hironori Tanaka, Akitoshi Hakoda, Noriaki Sugawara, Kazuhiro Ota, Hiroki Nishikawa

**Affiliations:** ^1^ Gastroenterology Center Hirakata Municipal Hospital Osaka Japan; ^2^ Second Department of Internal Medicine Osaka Medical and Pharmaceutical University Osaka Japan; ^3^ Endoscopy Center Osaka Medical and Pharmaceutical University Hospital Osaka Japan

**Keywords:** esophageal endoscopic submucosal dissection, esophageal fully covered self‐expandable metallic stent, perforation, stent‐anchoring method, superficial esophageal neoplasm

## Abstract

Patients with esophageal endoscopic submucosal dissection‐associated adverse events can have a severe clinical course. In this report, we describe the case of a 67‐year‐old male with a history of endoscopic submucosal dissection who had metachronous superficial esophageal cancer. Although endoscopic submucosal dissection was attempted for challenging lesions adjacent to the scar, perforations occurred during the procedure. We placed an esophageal fully covered self‐expandable metallic stent at the perforation site to avoid emergency surgery after lesion removal. However, stent migration and displacement concerns remained, as no stenosis was observed in the esophagus. Therefore, a clip with a thread on the upper edge of the stent was placed, and this anchoring clip was useful in preventing stent migration and closing the perforation. The patient improved with conservative treatment. In conclusion, the stent placement and anchoring method with clip and thread could be a treatment option in such cases.

## INTRODUCTION

Superficial esophageal neoplasms with a low risk of lymph node metastasis are good indications for endoscopic submucosal dissection (ESD). Although ESD is a minimally invasive treatment, patients with post‐ESD complications, including delayed bleeding and perforations, are likely to follow a severe clinical course. Perforations, which can occur unexpectedly, may have a severe or fatal prognosis. Therefore, establishing feasible methods to manage ESD‐associated complications is necessary. We report a case of esophageal perforation during ESD.

## CASE REPORT

**FIGURE 1:**
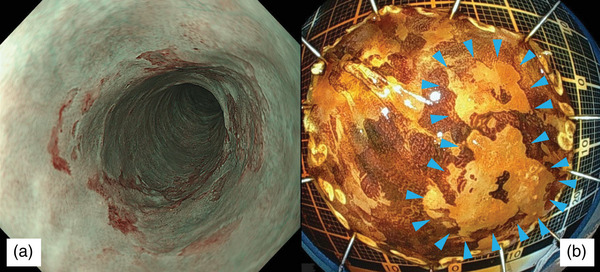
(a) Endoscopic images of the superficial esophageal cancer with narrow‐band imaging. The lesion is located at the thoracic esophagus 26 cm from the incisors near the previous scar after endoscopic submucosal dissection. (b) Endoscopic images of resection specimen with iodine staining. The resection specimen revealed a lesion 20 mm in size (Blue arrows represent the lesion).

**FIGURE 2:**
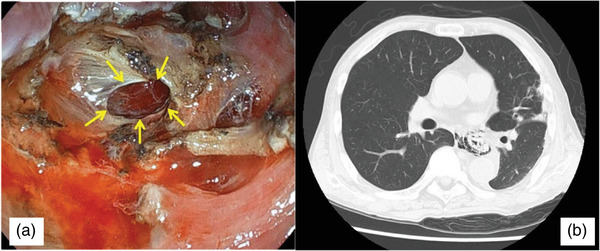
(a) Endoscopic images of the perforation (yellow arrows). The esophageal muscle layer is damaged and perforated during endoscopic submucosal dissection. (b) Computed tomography scan shows free air in the mediastinum of the thoracic and abdominal esophagus.

**FIGURE 3:**
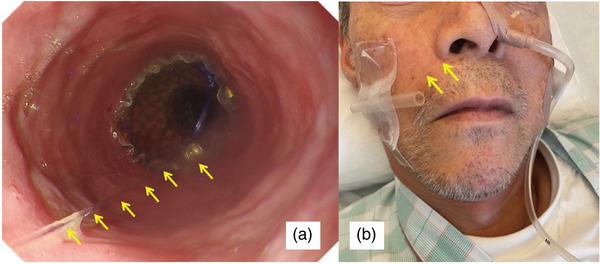
(a) Endoscopic image of the esophagus after the endoscopic stent placement on the perforation. The fully covered self‐expandable metallic stent was fixed with an anchoring clip and thread (yellow arrows). (b) The thread (yellow arrows) and drainage tube were fixed in the right and left nostril holes, respectively.

**FIGURE 4:**
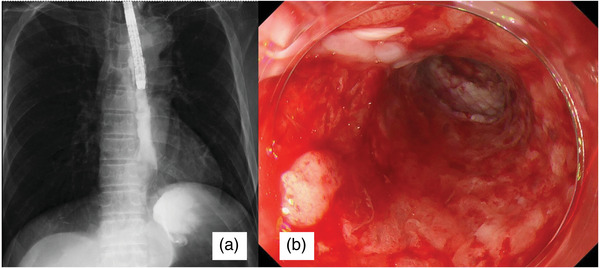
(a) Radiographic images with contrast agents showing no leakage after removing the covered stent. (b) Endoscopic image of the esophagus after stent removal. No perforation is observed on the ulcer induced by endoscopic submucosal dissection.

A 67‐year‐old male had a history of ESD for thoracic esophageal cancer approximately 15 years ago. The patient also had a history of rectal cancer, and a computed tomography (scan performed during the follow‐up revealed a thick cervical esophageal wall. Therefore, esophagogastroduodenoscopy (EGD) was performed, revealing Stage IVB advanced esophageal cancer in the cervical esophagus 20 cm from the incisors and subcircumferential superficial esophageal cancer in the thoracic esophagus 26 cm from the incisors near the previous ESD scar. Cervical esophageal cancer was treated with priority, considering the patient's prognosis, and definitive chemoradiotherapy was performed half a year ago. The region of radiotherapy was limited to the cervical esophagus because only cervical esophageal cancer was considered a prognostic factor. EGD was performed to confirm the chemoradiotherapy's effectiveness. The cervical esophageal cancer responded to the treatment. Additionally, the volume of thoracic esophageal cancer was reduced, although it persisted. The superficial esophageal cancer was at the thoracic esophagus 26 cm from the incisors, 20 mm in size, and morphologically 0‐IIa+0‐IIb (Figure 1a,b). Histopathological examination of the biopsy specimen revealed squamous cell carcinoma and the tumor was suspected to be confined to the mucosa. Therefore, we resected the lesion endoscopically, although this was challenging because of the presence of a scar after the previous ESD. We used a therapeutic endoscope (EG‐840TP; Fujifilm Medical) and endoknives (IT nano; Olympus Medical Systems Corporation, and Flushknife BT 1.5 mm; Fujifilm Medical) for the esophageal ESD. However, ESD for scar lesions after a previous ESD induced submucosal fibrosis and made dissection difficult. A perforation occurred on the left side of the mucosal defect during the procedure (Figure 2a) but was closed using clips (Sure Clip, 8 mm; Micro‐Tech) after lesion resection. However, closing the perforation was difficult because the muscle layer was fragile, and the clip's edge caused the perforation to tear. Computed tomography scan revealed free air in the mediastinum of the thoracic and abdominal esophagus but no subcutaneous emphysema (Figure 2b). A covered metallic stent (HANAROSTENT, 100 × 18 mm; Boston Scientific) was endoscopically placed in the thoracic esophagus to close the perforation. However, stent migration or displacement was a concern in the absence of esophageal stenosis. Therefore, we placed a clip with a nylon thread (fishline) on the stent's upper edge to prevent displacement toward the anal side (Figure 3a). The clip with the thread was fixed solely to the stent with an endoscope. Next, the thread was removed from the mouth while the endoscope was withdrawn. A Nelaton catheter was inserted through the right nostril to the mouth, and the thread was tied to the catheter. The catheter was removed from the nose, and the thread was secured to avoid shifting. Furthermore, the patient's nostrils were examined, and the intraesophageal drainage tube was fixed in the left nostril (Figure 3b). Antibiotics were administered from the day of perforation. C‐reactive protein level increased to 8.12 mg/dL the following day. No apparent fever was observed post‐treatment. After 9 days, the C‐reactive protein level improved to 1.81 mg/dL, and leukocytes were normalized, leading to the termination of antibiotic treatment. Mild discomfort in the chest was present, but no obvious chest or abdominal pain. One week later, a computed tomography scan revealed a decrease in mediastinal emphysema. The esophageal stent was endoscopically removed on day 17 after placement, and the clip attached to the stent was confirmed to be present. No perforation was observed in the artificial ulcers. Radiographs with contrast agents did not show leaks (Figure 4), and the patient resumed eating on the same day. Endoscopy revealed no perforation 5 days after stent removal, and the patient was discharged from the hospital.

Histopathologically, the resected specimen revealed esophageal squamous cell carcinoma confined to the lamina propria without lymphovascular invasion and with negative margins. Complete resection was achieved.

## DISCUSSION

ESD is a minimally invasive treatment; however, the associated adverse events, including bleeding, perforation, and stenosis, cannot be completely avoided. In the real world, the incidence of perforation during ESD is significantly low at approximately 1.9%.^1^ Here, we experienced a successful case of using a fully covered self‐expandable metallic stent (FCSEMS) for perforation closure during esophageal ESD to avoid surgery.

Most of the small iatrogenic perforations improve with conservative treatment.^2^ However, some cases can take a severe clinical course. Endoscopic and surgical treatments initiated within 24 h after a spontaneous or iatrogenic esophageal perforation result in a mortality rate of 7.4% compared to 20.3% in patients who are treated later.^3^ Therefore, in cases with stable vital signs, endoscopic closure should be considered first for perforation, which has various methods, including through‐the‐scope clips, over‐the‐scope clips (OTSC), and FCSEMS.^4^ Through‐the‐scope clips are effective for closing small defects; however, if the muscle layer is highly fibrotic or exposed, clipping is ineffective and leads to further tearing. OTSCs enable larger defects to be closed, but their placement in the esophagus is a little difficult because of the narrow space. Therefore, closure with FCSEMS might be effective for large perforations that are difficult to clip in the esophagus.

The difficulty of esophageal ESD is influenced by the following factors: lesion sizes >30 mm, the circumference of the lesion >1/2, and the location at the left side wall or the lower esophagus.^5^ ESD for lesions with scars generally has a higher incidence of perforation than for those without scarring.^6^ Several types of esophageal stents are available. The placement success rate between partially covered SEMS and FCSMES is similar.^7^ Additionally, the mortality rate of patients with esophageal perforation who underwent SEMS management was 7.3%.^3^ These cases involve perforations caused by iatrogenic diseases, foreign bodies, and malignant diseases.^3^ However, although SEMS is covered by insurance in Japan for the treatment of stenosis caused by malignant tumors, it is not covered by insurance for the closure of perforations. On the other hand, self‐expandable plastic stents are not approved by regulatory authorities in Japan, although they are used in other countries.^7^ SEMS are superior to self‐expandable plastic stents in terms of therapeutic efficacy, risk of deviation, and likelihood of stent migration. Migration or displacement may occur when stents are used for benign esophageal strictures; therefore, so in such cases, it is ideal to remove the stent after a short period of time, such as 1–2 weeks. This indicates that stents need to be fixed using endoscopic suturing or OTSC to prevent migration.^7^ The timing of stent removal is generally decided based on the improvement of mediastinal emphysema and mediastinitis. In the present case, the stent was removed after 17 days because it was just after the holiday season. Although the time of removal was delayed, the anchoring clip maintained the stent location 17 days later. Stent migration occurs in 15.9% of cases, even with endoscopic fixation after stenting.^8^ The tension of the thread fixed to the face via the nostril could be useful for assessing the displacement. According to one report, colonic stents (24 mm) are thicker than esophageal stents and thick metal stents are effective in preventing stent dropout.^9^ A similar conservative treatment involving stenting and anchoring clips was used for Boerhaave syndrome.^10^ Based on this report, we adapted the same method, which proved effective in such cases. This strategy is useful when a perforation cannot be closed using clips. Additionally, our method requires only endo‐clips and thread, making it a more cost‐effective option than other methods for stent fixation, such as suturing or OTSC.

In conclusion, the stent anchoring method using clip and thread for iatrogenic perforation associated with esophageal ESD could be considered a viable option to avoid emergency surgery.

## CONFLICT OF INTEREST STATEMENT

None.

## CLINICAL TRIAL REGISTRATION

N/A
